# How does a Network Platform Work for Participating Actors Towards Integrated Care Governance? A Case Study of a Dutch Hospital Region

**DOI:** 10.5334/ijic.6736

**Published:** 2022-12-20

**Authors:** Oemar van der Woerd, Elizabeth van Veen-Berkx, Wilma van der Scheer, Roland Bal

**Affiliations:** 1 Erasmus School of Health Policy & Management, Erasmus University Rotterdam, Burgemeester Oudlaan 50, 3062 PA Rotterdam, NL; 2 Erasmus Centre for Healthcare Management, Erasmus University Rotterdam, Burgemeester Oudlaan 50, 3062 PA Rotterdam, NL

**Keywords:** integrated care governance, network platforms, physicians, hospital directors, qualitative research

## Abstract

**Introduction:**

Network platforms are interesting for integrated care governance as they seek solutions for the problem of competition and tensions between networks. In this paper, we analyse how a network platform functions for the actors involved, and how it is used in their work.

**Methods:**

We employed a case study in a Dutch urbanised hospital region, and conducted 17 interviews with hospital physicians, directors, and supporting staff who are involved in a network platform called “BeterKeten” (BK).

**Results:**

Actors assign different functions and purposes to BK: facilitating and legitimising professional (learning) communities; adapting to a changing policy context; enlarging professionals’ and the networks’ circle of influence; and extending governing possibilities. Network platform’ dynamics and frictions entail changing professional and managerial practices; embedding a BK network in a partner network; and alignment of (conflicting) network platforms.

**Discussion:**

Network platforms are a promising strategy to govern, facilitate, and nurture network-building actions to enhance integrated care, offering new ways of working to cope with its multi-level nature.

**Conclusion:**

BK is a dynamic actor with steering capacities that enables the co-existence of multiple purposes. Further research could pay attention to how network platforms are able to develop modalities of integrated care governance that suit healthcare system’s networked character.

## Introduction

Researchers and practitioners are increasingly addressing the networked and multi-level nature of integrated care to develop suitable governance within (fragmented) health and social care systems [1,2,3,4]. Collaborative governance—including engaging different stakeholders while building trust—is identified as a working mechanism for integrated care [1]. Inter-organisational networks, understood as collaborations amongst multiple healthcare organisations and professionals [5], are widely accepted and used for the coordination of health and care services to meet patients’ needs [4].

Literature on integrated care governance predominantly focuses on governance structures and configurations within *individual* networks, and to what extent these conditions influence network effectiveness—understood as networks reaching their objectives [6,7]. Scholars have developed frameworks for the establishment, governance, and evaluation of individual networks [8,9]. Illustrative of this focus are the distinguished forms of integrated care governance [10]: *a* network coordinated by a separate entity; *a* network governed by a leading organisation; or organisations that jointly govern *a* network. This, however, conflicts with the empirical realities of professionals and organisations who are increasingly enmeshed in a web of *multiple* inter-organisational networks [11]. Networks may overlap, interact, and possibly compete in terms of goal-setting [12], leading to pressures on professionals’ agendas or moral dilemmas about in which network to participate as time and financial resources are limited [13]. Though network variety in terms of characteristics is acknowledged [6], attention towards the multiplicity of networks for integrated care governance remains scarce. The question becomes not necessarily how to effectively govern an individual network, but how to navigate multi-network dynamics to enhance integrated care.

Scholars of network governance describe network and collaborative platforms as a centralised and external form of governance to facilitate, enable, and regulate distributed network-building actions [12,14]. Network platforms are interesting as they seek a solution for the problem of competition and tensions between networks. In this paper, we analyse a network platform called “BeterKeten” (BK), operating in an urbanised southwestern region of the Netherlands, which houses multiple inter-organisational networks and has succeeded in establishing both “horizontal” integration (i.e., coordination and shared clinical services amongst hospitals), and “vertical” integration (i.e., hospital services with community and primary care) in the last decade [15]. We are interested in how this platform works “from within” [16]—that is, how it is actually used in constituent actors’ work practices. We aim to specify how a network platform can sustain network-building actions to enhance integrated care governance. In this, we apply an interpretative and dynamic approach to network platforms by focusing on the experiences and strategies of actors involved in BK (i.e., hospital directors, physicians and BK staff). Interpretative research into what a network platform means for participating actors, and how they make use of it, may foster a fine-grained understanding of how multiple networks are governed, and with what dynamics and frictions [17]. This may challenge and reconsider rather general and abstract notions of network governance [8]. Being aware that it is a great challenge to capture the complexities of the multiplicity of networks for integrated care governance, we narrow down our focus to the perspectives of *participating* actors. The dynamics and frictions we unravel emerging through and within BK hence offers a partial yet important account of how a network platform works towards integrated care governance. The following research question guides our analysis: *How does a network platform work towards integrated care governance from the perceptions of participating actors, and which frictions and dynamics emerge through and within the network platform?*

To develop an understanding of the practices of and within the network platform, we conducted interviews with hospital physicians and directors who are involved in four (of the 21) clinical networks housed by BK. We used (non)participant observational notes of several BK gatherings to complement our data. Qualitative input about how a network platform functions for actors involved may offer valuable insights for those charged with shaping integrated care governance [18].

## The enabling role of network platforms

A network platform is defined as “an organization or program with dedicated competences, institutions and resources for facilitating the creation, adaption and success of multiple or ongoing collaborative projects or networks” [14, p.20]. “Network administrative organizations” (NAOs)—a separate entity that coordinates network actions—are often described as a specific governance structure to govern *a* network [8]. A network platform may enable the (re)organisation of network-building actions in response to a changing context [19]. Also, platforms mediate between local networks and national authorities [14], facilitating change beyond network boundaries, for example at health-system levels [12]. Furthermore, interactions among networks within the platform may create learning opportunities around how to network or enlarge a network’s focus in the wider healthcare context [12].

With these enabling functions potentially leading to synergies between network-building actions, network platforms are seen as a specialised mode, strategy, or mechanism to cope with network-level tensions [8,20]. First, network platforms encourage inclusiveness by allowing actors to participate in governance processes, whilst focusing on achieving their networks’ objectives. Second, collaborative processes strengthen internal legitimacy, whilst network actors may represent the network to others to obtain external legitimacy. Third, network platforms nurture a sense of unity amongst actors, whilst maintaining actors’ diversity.

These tensions mainly relate to individual-network levels, thus overlooking how multi-network dynamics enable or constrain actors’ work practices. Also, they seemingly assume a rather singular purpose in a network platform, restricted to governance matters. This may additionally overlook the diversity of platform functions, and the bottom-up dynamics and frictions between and within network platforms. For instance, the layering of multiple policy initiatives for integrated care may not only hinder actors’ network-building on a day-to-day basis [13], but could also cause confusion for a network platform regarding what to focus on. Furthermore, diverging interests and resources among network actors influence the evolution of a network platform [21]. Others have shown that network actors use power strategies to deal with other members’ opposing views, leading to less-inclusive platform types [22].

In this study, we conceptualize a network platform as a dynamic entity that may enable and fulfil *different* functions for *different* actors that moreover may *change* over time [23]. To analyse how the central actors—hospital directors, physicians and BK staff—perceive the network platform and for which purposes and strategies, an interpretative perspective is needed, paying attention to actors’ perceptions of how they use the platform in their work [24]. In doing so, our paper further builds on literature that critically analyses collaborative forms of governance (in healthcare) by zooming in on emerging actor-level tensions, and how these inform our understanding of how a network platform ‘works’ [17,23,25]. With network-building we refer to the intentional and ongoing efforts of participating actors to create, nurture and sustain networks [26,27]. These include the development of relationships beyond organizational boundaries or efforts to integrate care delivery processes to shape a networked model of care. For the purpose of this study, we define integrated care as:

“[…] a coherent set of methods and models on the funding, administrative, organizational, service delivery and clinical levels designed to create connectivity, alignment and collaboration within and between the cure and care sectors. The goal of these methods and models is to enhance quality of care and quality of life, consumer satisfaction and system efficiency for patients […] cutting across multiple services, providers and settings. [Where] the result of such multi-pronged efforts to promote integration [lead to] the benefit of patient groups [the outcome can be] called ‘integrated care’” [28, p. 3].

This definition fits with our scope as it highlights inter-organisational collaboration on various levels, including clinical ones, to enhance integrated care.

## Methods

### Research background: The BeterKeten network platform

**Figure 1:**
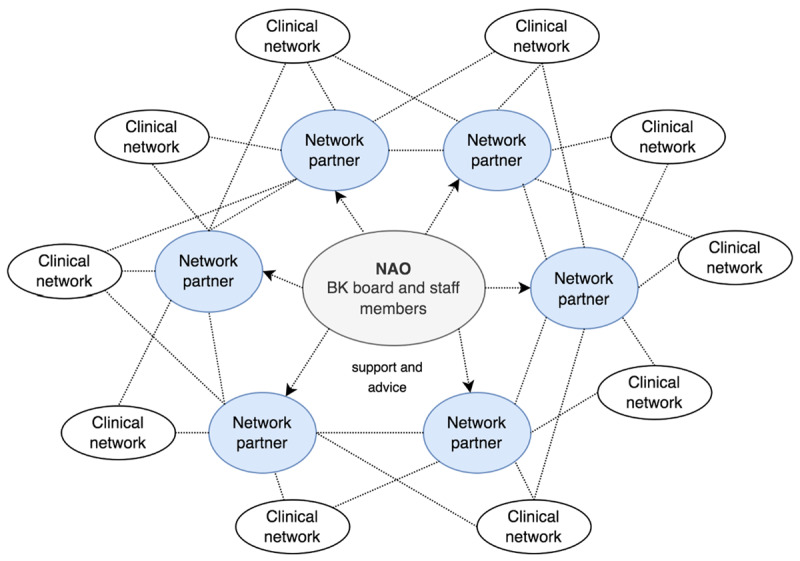
Simplified visualization of the BK network platform.

In the last decade in the Netherlands, several hospital initiatives at regional and national levels have been initiated to cluster clinical expertise in order to develop clinical pathways for specific diseases like thyroid and oncology care [29]. One of those initiatives is BK, established as a foundation (legal form) in 2011 by two hospitals in the Rijnmond region to enhance overall hospital care by initiating inter-organisational projects like aligning patient referral systems. Figure 1 visualizes how BK is structured. BK is the name of the NAO that governs a network of clinical networks. These clinical networks have separate names, but are clustered in BK. The BK board consists of a selection of hospital directors and physicians who work for one of the by now six affiliated network partners (i.e., hospitals in the Rijnmond region). The chair rotates among the partners. Each clinical network has a separate board that supervises its day-to-day functioning, and organizes gatherings to discuss disease-specific matters. BK staff temporarily support the clinical networks with project and management expertise regarding network-building and the organisation of clinical research, and organize gatherings for network partners to discuss healthcare developments. BK staff hence does not initiate clinical networks by *themselves*, but nascent clinical networks can request support from BK. BK staff visit network partners to monitor or further develop network-building actions and ambitions, and consult them about network opportunities. Once a clinical network flourishes, BK staff intends to withdraw, leaving the network to its own functioning, unless organizational issues arise. To date, 35 clinical projects (including 21 clinical networks) and 11 PhD trajectories have been initiated involving approximately 500 hospital physicians. BK is interesting to study as a network platform because it houses multiple clinical networks (with most networks still actively supported), and has successfully operated in the healthcare sector for more than 10 years.

### Data collection

Following a case study methodology, we interviewed actors that work for BK (i.e., BK staff) and actors that are involved in the clinical networks supported by BK (i.e., hospital directors and physicians) to explore how BK functions and affects their work practices. Between April and September 2020, we conducted 17 semi-structured interviews: 12 interviews with hospital physicians, four interviews in pairs of hospital directors and medical staff directors working in the same hospital, and an interview with the former director of BK. These respondents were selected because they are involved in four clinical networks within BK: (1) *Obesity Centre CGG*, (2) *Thyroid Network*, (3) *Pediatric Rheumatology Network*, and (4) *Partners Gynecology, Obstetrics and Reproductive Medicine*. We interviewed three associated physicians, the hospital director, and the medical staff director of each network. The representation of respondents reflected the variety of included clinical networks, and helped to understand the development and working of BK more precisely. These networks were selected based on diversity in years of existence (i.e., initiated in the beginning years of BK or more recently); network scope and composition (i.e., different clinical specialties, and involvement of other healthcare organisations); and type of activities (e.g., specialised clinical services or integrated pathways with community services). During these in-depth interviews, we asked respondents to reflect on the network platform: Why and how does BK work? How does it affect (or not) their work practices? What future directions should the platform explore? We asked respondents for real-life examples that illustrate how specific dynamics or characteristics of the network they are involved in enabled how they made use of BK.

Interviews were conducted in-person or digitally due to COVID-19 measures limiting physical access to hospitals. The interviews each lasted 45 to 60 minutes. Audio recordings were transcribed shortly after the interviews had occurred, with permission from all respondents. To ensure that the interpretations reflected the understandings of respondents, the first and second author presented preliminary findings during two online BK board meetings (October 2020 and February 2021). We made observational notes in which we wrote down reflections of attendees. Nonparticipant observations were made during a BK network platform gathering mid-2020 to become familiar with BK’s way of working. The discussions and notes helped refine the data, bolstering an iterative process of member-checking and triangulation to validate findings.

### Data analysis

We abductively analysed the interviews using Atlas.ti software [30]. We iteratively coded the interviews, going back and forth between data and theory about network platforms and governance. We first derived themes related to why and how BK works: shared ambitions for clinical excellence and research; possibilities to learn from peers; and reciprocity while collaborating. These themes helped us organise the data, and were then analysed more precisely by zooming in on actors’ experiences with BK. This led to the four themes presented in the first results section. Informed by theory on network platforms, we analysed the interview transcripts again while paying attention to emerging dynamics and frictions between platform layers (i.e., physicians, directors, BK staff), and between BK and its context (i.e., surrounding platforms and stakeholders). This led to the three themes presented in the second result section.

## Diverging functions of a network platform

### Facilitating and legitimising professional (learning) communities

BK enables physicians to develop (informal) professional communities that find common ambitions for clinical excellence. For them, BK is a learning platform for timely and informal access to other physicians, execution of clinical research, and development of expertise. For patients, these communities result in a widely supported assessment of their care needs as multiple opinions are discussed in a clinical network [31]. Such professional communities are often led by renowned physicians who adopt a leading role in network-building. Professional relationships enable BK to exist, but formalising and “labelling” these relationships as a BK initiative helps physicians to receive support for which they themselves do not have the expertise and time:

“The Thyroid Network would never have started without BK, because now we have a club that facilitates and thinks along professionally, and functions as a bridge between network participants. I don’t think participants in our network could have done this on their own. We didn’t have the workforce, time and expertise. BK has been and still is of vital importance to our network. The professionalism and cohesion will otherwise be lost.” (Hospital physician, interview)

BK’s labelling and support acknowledges that physicians’ network-building actions matter for (the organisation of) clinical integration, legitimising their role in network-building. Moreover, it facilitates and eases day-to-day learning beyond one’s individual work practices.

### Adapting to a changing policy context

BK also offers participating actors somewhere to process and adapt to a changing and increasingly complex healthcare policy context [32]. BK was primarily initiated in response to quality criteria and volume standards imposed by professional associations, against the backdrop of national policy discussions about the role of academic centres and other specialised hospitals [29]. This development spurred hospitals in Rijnmond to deliberately cluster surgical operations. BK staff guided physicians in building clinical pathways to follow quality regulations. The role and scope of BK, however, has incrementally changed in the last decade due to policy developments. Alongside a focus on quality regulations, cooperative strategies like “regionalization” to handle capacity shortages [33] are emphasised within a healthcare context of regulated competition [34]. BK has thus evolved into a platform that focuses on a broader set of questions involving peripheral hospitals and other healthcare organisations. This is illustrated in how the Obesity Clinic CGG evolved over time:

“In 2009, we [obesity care physicians] asked to the Board of Directors: ‘Do we consider obesity an academic theme?’ […] We thought it was, because it was a social problem. We wanted to offer a treatment trajectory for obesity patients. We, however, did not have an important treatment part, bariatrics, because of limited operating capacity. […] BK played an important role in making obesity a legalized academic topic. Through collaboration with other hospitals, we learned how to recognize patient needs, and were able to link care to clinical research. BK allowed us to offer patients suitable treatments by dividing those amongst the academic and peripheral hospitals. […] In our pathway, we now have debt counseling and neighborhood coaches for light care needs, up to more specialized care.” (Hospital physician, interview)

Though the Obesity Clinic CGG started with physicians, it became an integrated care pathway with professionals working in healthcare and welfare domains. BK staff helped physicians to materialise their ideas on how to enhance integrated care for patients by successfully engaging other hospitals, primary care, and the municipality to align obesity care trajectories. For network actors, BK provides a foundation to work from, gradually expanding the networks’ scope from clinical-orientation to integrated care.

### Enlarging professionals’ and the networks’ circle of influence

BK enlarges professionals’ and the clinical networks’ circle of influence, thus it is used as a vehicle for cross-network interactions with national policy-making layers to enhance integrated care [32]. Involvement with BK strengthens professionals’ positions during negotiations with external stakeholders like the Ministry of Health (MoH) and health insurers to achieve goals beyond hospital boundaries:

“Our network is taken more seriously during negotiations with the MoH. […] We have initiated the ‘combined lifestyle intervention’ [CLI], which is a strength of the Obesity Clinic CGG. I helped the MoH and the Care Institute [which advises the MoH on the insurance basket] to include the CLI in the basic insurance. […] We have presented patient narratives, quality of life effects and potential costs savings. Then the ball started rolling, and since January 2019 the CLI is reimbursed. This sounds like an easy story, but we have monthly meetings with the MoH about financial barriers and dysfunctional referral systems.” (Hospital physician, interview)

The MoH moreover considers the Obesity Clinic CGG a leading partner in developing a nation-wide integrated approach for obesity care, because the network included three hospitals with different diagnostic expertise. BK enables networks to work across hospital boundaries and the “place” they were primarily intended for (the Rijnmond region), strengthening strategic positions in the healthcare system to overcome institutional obstacles regarding integrated care. Hence, BK functions for physicians as an intermediate between healthcare and policy [12], allowing them to accomplish change on the system-level in order to enhance integrated care. This is referred to as “networking beyond the network” [12].

### Extending governing possibilities

Lastly, BK creates an “outside” governance layer for network actors, which extends their governing repertoire beyond its normal intra-organisational scope. This governance layer accommodates interactions between directors and physicians who work in different hospitals, as well as with BK staff. BK creates a possibility to link the scope of clinical networks to organisational interests. For instance, through the work of BK staff, the bottom-up network initiatives amongst physicians are distilled and made “visible” for hospital directors. This allows directors to consider network involvement, if desirable and possible, and to make organisational interests part of BK’s still-undefined future directions. BK is also referred to during national meetings by hospital directors to accomplish change within the organisation:

“I’m going to the NVZ and the STZ [national hospital associations], so they can say: in our region [Rijnmond], they have organized a pathway together, and that’s where you have to go as a patient. That also helps me within the hospital to say: if we can do that in the urology department, then we can also do that in other hospital departments.” (Hospital director, interview)

On the one hand, network actors see BK as a separate entity (i.e., a NAO) that allows cross-organisational and cross-network interactions. The examples above also indicate that BK provides a more fluid infrastructure to pragmatically pursue professional and organisational interests. This form of flexible governance cannot be acquired from intra-organisational governance positions alone.

## Network platform frictions and dynamics

### Changing professional and managerial practices

BK allows physicians and directors to involve themselves in each other’s work practices. For instance, imposed quality criteria and volume standards make cooperation amongst gynaecology and thyroid physicians more-or-less inevitable. For hospital directors, BK is another route to stimulate physicians to network. Directors therefore promote BK within the organisation as not all physicians realise what BK has to offer them: “BK is for many physicians one of the many logos. They do not know what is behind the logo” (physician, interview). BK staff deciding which projects will receive support can be seen as a means of steering *where* to network. Physicians are involved through BK in managerial practice: they use the multi-level nature of networks to accomplish change outside the realm of intra-organisational positions. Integrating network-building in daily work is, however, cumbersome as it adds to an already heavy patient-related workload:

“Multidisciplinary consultations on regional level are central to our network. However, unlike other consultations within our hospital, we organize those regional consultations at the end of the working day. We then plan two hours to discuss complex patient cases. I think it’s amazing that people put in this energy, but it also makes a working day extremely long.” (Hospital physician, interview)

Regional consultations allow for knowledge exchange opportunities, but also affect physicians’ work-life balance. Physicians question how network-building can be classified as patient-related work, illustrating how existing work practices are subject to change.

### Embedding BK networks within network partners

Though BK staff ease physicians’ work pressure with project and management expertise, its support is temporal. This is because of limited BK staff, but also based on the conviction of BK board members that at some point networks must continue independently, hereby nurturing network-building as an integrated part of professional work in the long-term. Hospital directors and BK staff find it difficult to decide when and how to embed a clinical network established under the BK flag within a network partner (i.e., hospitals) that functions as leading actor. They suggest giving more responsibilities to physicians as they actually shape clinical networks in their everyday work, and could thus most likely contribute to network sustainability:

“How are we going to manage this [physicians’ network-building actions], and how to keep it governable? We are now in many meetings, with a sandwich on the side, with 8 or 10 people. But do we dare to mandate a smaller group to get more things done? Because growing and doing more with the same board, I think that’s utopian.” (Hospital director, interview)

Delegating far-reaching responsibilities for network sustainability to physicians is difficult as directors are responsible for overall hospital performance and thus also the organisations’ involvement in networks. Organisation-centred regulatory frameworks moreover prevent the undertaking of network-level responsibilities.

### Aligning (conflicting) network platforms

While BK primarily focuses on clinical care and research, it has gradually broadened its scope in recent years to other care-related subjects requiring the involvement of peripheral hospitals. As a result, BK began to interfere with other regional and national platforms in the field of healthcare governance. These platforms exist side-by-side, and overlap in terms of scope (e.g., hospital care or closely related) and purpose (e.g., caring for regional populations). Hospital directors (among others) therefore question how competing platforms can be made productive:

“You want to achieve your goals as a hospital, but also as a region. I think that the cohesion of BK, SRZ, RotterdamseZorg, Regiovisie, Zorgdelta [all network platforms in the region] can be improved. We have to ensure that all those initiatives are aligned. For instance, within BK, we would like to guide patients during pregnancy for integrated gynecological care. We could use RotterdamseZorg to train professionals who work through administrative boundaries.” (Hospital director, interview)

Though BK coordinates network-building actions on a regional level, it also comes with the new challenge of aligning conflicting platforms. This is cumbersome; each platform has its own governance structure and relates to different health and welfare domains with other laws and regulations. Moreover, ideas about the platform’s future direction differ. Some participating actors would like to see that BK further extends its purpose to cooperate more intensively with primary care. Others are more conservative, arguing that BK should remain focused on clinical integration to prevent unmet ambitions. Hence, co-existing platforms with similar aims urge participating actors to rethink BK’s identity and boundaries to remain distinctive in an increasing networked healthcare context.

## Discussion

Forms of integrated care governance predominantly focus on how to effectively govern an individual network. However, how to navigate through multiple networks simultaneously receives relatively little attention. Network platforms are therefore interesting for integrated care governance as they seek solutions to the problem of competition and tensions between networks. Following an interpretative perspective [24], this paper analysed a network platform (BK) in a Dutch hospital sector that houses multiple clinical networks, exploring how a network platform works and affects constituent actors’ work practices.

Our results show that a network platform is important for actors for a variety of reasons: it facilitates and legitimises professional (learning) communities; it helps to adapt to a changing policy context; it enlarges professionals’ and the networks’ circle of influence; and it extends governing possibilities. However, emphasis on and opportunities for network-building in the BK context change professional and managerial practices, for instance by putting pressure on their work-life balance. Embedding a BK network in a network partner primarily led by hospitals (and not by BK staff) is moreover considered difficult as responsibilities primarily lie at the hospital organisational level. Furthermore, co-existing network platforms may conflict, and require alignment to prevent over-complicated integrated care governance.

BK provides a structure for governing increasing network-building actions that are scattered across the region [8], but also dynamism, as it functions as a vehicle for a variety of purposes for multiple agents. This was for instance shown in how physicians use the network platform to shape professional learning communities, but also for cross-organisational and cross-network impact regarding integrated care. We distil several enabling capacities of a network platform. First, it can reorder existing care delivery activities and priorities of network actors in a changing regulatory context (*purpose-rearrangement*). Our case illustrated that BK enabled hospital directors and physicians to develop clinical pathways to follow quality regulations. Second, it may turn away from old focuses of network actors or an individual network towards new strategic ones (*purpose-reorientation*). BK for example enabled hospital directors to give shape to regionally-oriented healthcare policy, and to reconfigure the organizations’ position accordingly. Third, it allows for the exploration of new types of network-building actions, incrementally building on existing achievements generated from previous network-building actions (*purpose-extension*). BK enabled network actors to explore new organizational arrangements with professionals operating in *other* healthcare and welfare domains, and to bring about far-reaching policy changes to enhance integrated care. Besides context-driven (policy) reasons that call for more integration beyond clinical services, a network platform’s rearrangement, reorientation, or extension is made possible through deliberate actor-level work. Network actors in our case recognised, created, and used the flexibility of BK in their work practices. This indicates “function creep” of a network platform that is purposive, understood as the gradual expansion of the networks’ functions beyond what they were originally created for [35]. BK started to focus on clinical integration, but gradually became a place for broader and underexplored questions regarding integrated care to accommodate complex patient needs [31]. The platforms’ function creep could be an explanation why BK interferes with other surrounding network platforms. This seems inherent to network platforms that function as an enabler for various purposes, making it more likely to overlap and collide with others.

Because our study aimed to develop a more precise understanding of what a network platform means for network actors, and how it is used in their work, we selected respondents who are strongly familiar with BK (i.e., involved in the four selected clinical networks). A limitation of our study is that we only analysed an individual network platform in an urbanised region, with the possible consequence that we were unable to compare network platform functions and frictions in other healthcare and welfare domains or less-urbanised areas.

Our study provides several implications for the networked and multi-level nature of integrated care governance [36]. First, network platforms are a promising strategy to govern, facilitate, and nurture network-building actions to enhance integrated care. Our results may serve as input for practitioners and policymakers to build and further craft network platforms for integrated care governance [18], for instance how to utilise professionals’ ambitions and expertise for (the organisation of) clinical care and research. Though interfering network platforms could be made productive through alignment, this should warn policymakers and practitioners about the consequences and feasibility of network governance for everyday practice. Second and related, network-building as an integral part of professional work cannot be taken for granted; to be sustainable, it requires caring for network actors by organising support when a network is established or considered operational. The identified network platform’ dynamics and frictions should challenge our thinking as to what extent network-building is part of healthcare governance work, with what responsibilities, as well as how it can be extended beyond only a select group of renowned physicians. Working in networks may be useful, but also asks of network actors to develop strategies that make a networked healthcare context less complex. An example involves the mobilisation of policy actors to bring about institutional change. Third, network platforms’ mediating role between shopfloor, organisational, and policy levels accommodates change for integrated care policies across individual network boundaries. Network platforms therefore offer new and unexpected ways of working to cope with the multi-level nature of integrated care.

## Concluding remarks

We conclude that the functioning of a network platform cannot be reduced to primarily governance purposes. Our interpretative analysis of a network platform in a Dutch hospital region shows that different actors assign different, co-existing functions and purposes to BK. These are related to clinical integration, but also those that cut across intended platform aims like “external” governing possibilities and health-system impact. BK is more than the sum of collaborating hospitals that together with their clinical networks form a “network of networks” [12] that aim for integrated care across hospital settings. More precisely—BK is a dynamic actor with steering capacities that enables the co-existence of multiple purposes, including those related to governance, coordination, and the fulfilment of professional and organisational interests. How a network platform works hence depends on which actor perspective is taken. This highlights to integrated care governance studies the importance of including a variety of actors that operate on different policy-making layers to account for the multi-level nature of networks. Our study moreover informs integrated care governance studies by foregrounding how a network platform affects constituent actors’ work practices, and how actors *work with* a governance structure to enhance integrated care. It seems therefore worthwhile to further investigate how governance structures are perceived in actors’ work practices in other healthcare and welfare domains to shape suitably integrated care governance. Further research could especially study how network platforms are able to develop modalities of integrated care governance (e.g., supervision, accountability procedures, and leadership) that suits healthcare’s networked universe.

## Author Information

**Oemar van der Woerd** has completed MSc degrees in healthcare management and public administration and is currently working as a PhD candidate in the Department of Healthcare Governance at Erasmus University Rotterdam. Through mainly ethnographic research methods, his research focuses on the meaning and practice of governing across traditional organisational boundaries for healthcare organisations and their management.

**Elizabeth van Veen-Berkx** is currently working at the Netherlands Comprehensive Cancer Organisation (IKNL). She manages a project with the aim to innovate clinical trial designs by using real-time and real-world data. She has a broad work and research experience in healthcare management topics, ranging from inter-organisational collaboration, clinical leadership, and operating room scheduling.

**Wilma van der Scheer** is professor in healthcare governance and leadership and director of the Healthcare Management Centre at Erasmus University Rotterdam. She develops and teaches in several educational programmes for healthcare professionals, ranging from executives and managers to nurses. She wrote her dissertation on this issue, titled Under healthcare executives: Dealing with managerial ambiguity in healthcare (*Onder zorgbestuurders. Omgaan met bestuurlijke ambiguiteït in de zorg*, in Dutch).

**Roland Bal** is full professor of healthcare governance at Erasmus University Rotterdam. With a background in Science & Technology Studies, he is interested in the infrastructural work of layered healthcare settings. He mainly focuses on issues related to quality of care, and increasingly on the networked character of healthcare settings. He has published widely in STS, medical sociology, as well as public administration.
